# Air Sensor Data Unifier: R-Shiny Application

**DOI:** 10.3390/air3030021

**Published:** 2025-08-30

**Authors:** Karoline K. Barkjohn, Catherine Seppanen, Saravanan Arunachalam, Stephen Krabbe, Andrea L. Clements

**Affiliations:** 1 Office of Research and Development, United States Environmental Protection Agency, Research Triangle Park, Durham, NC 27711, USA; 2 Institute for the Environment, The University of North Carolina at Chapel Hill, Chapel Hill, NC 27516, USA; 3 Region 7, United States Environmental Protection Agency, Lenexa, KS 66219, USA

**Keywords:** air quality, air sensor, open source, R, data, format, standard format, reformat

## Abstract

Data is needed to understand local air quality, reduce exposure, and mitigate the negative impacts on human health. Measuring local air quality often requires a hybrid monitoring approach consisting of the national air monitoring network and one or more networks of air sensors. However, it can be challenging to combine this data to produce a consistent picture of air quality, largely because sensor data is produced in a variety of formats. Users may have difficulty reformatting, performing basic quality control steps, and using the data for their intended purpose. We developed an R-Shiny application that allows users to import text-based air sensor data, describe the format, perform basic quality control, and export the data to standard formats through a user-friendly interface. Format information can be saved to speed up the processing of additional sensors of the same type. This tool can be used by air quality professionals (e.g., state, local, Tribal air agency staff, consultants, researchers) to more efficiently work with data and perform further analysis in the Air Sensor Network Analysis Tool (ASNAT), Google Earth or Geographic Information System (GIS) programs, the Real Time Geospatial Data Viewer (RETIGO), or other applications they already use for air quality analysis and management.

## 1. Introduction

Poor air quality contributes to the burden of disease globally [1]. Air quality measurements are critical to provide information to adequately protect human health [2–5]. In addition to conventional air monitors, air sensors are becoming more widely used for a variety of applications [6,7]. These sensors are often lower in upfront cost, easier to site, straightforward to operate, and require less maintenance than conventional monitors, allowing them to be deployed by a wider range of users. Many networks contain dozens or more sensors reporting data every few minutes or faster.

Air sensors have been used to advance science, better understand local air quality, and further protect human health [8]. For example, PM_2.5_ sensors have been used to understand the impacts of short-term wildfire exposure on reduced attention span [9]. Air sensors can provide helpful information to make decisions about outdoor activities and indoor air pollution [10,11] and can help to better understand infiltration of outdoor particles into the indoor air [12]. Gas and particle air sensors have been used to determine source apportionment [13], develop emissions factors [14], and refine emission inventories [15].

Before drawing conclusions and protecting public health, users must perform an in-depth analysis of air sensor data [16]. Raw data reported by air sensor networks can have issues that require careful analysis to produce credible processed data [17]. Many sensors have biases out of the box and must be co-located or operated near conventional air monitors to understand performance and correct for any bias or the influence of relative humidity or other factors [18,19]. Analyzing and comparing a wide variety of datasets can be challenging due to large data volume, variable formats, and a variety of unique features and issues requiring extensive data analysis skills.

In the fall of 2019, the United States Environmental Protection Agency (U.S. EPA) staff conducted dialogs with U.S. EPA Regions, state, local, and Tribal air monitoring organizations to understand and document their technical needs associated with using air sensors and air sensor data as part of the “Air Sensors Data Dialogs” project [20]. These dialogs revealed technical challenges related to data management, analysis, and visualization, as well as the need for standardized data formats and increased technical information sharing. Subsequently, the “Air Sensor Data Solutions” project and report [21] outlined potential solutions to support these organizations that are increasingly using air sensor data but are also experiencing technical and capacity constraints. Potential solutions included data hosting support, establishing data quality objectives and indicators for sensors, code sharing, development support, and development of rapid data analysis and visualization tools. Potential near-term actions identified included the development of interoperable data formats and enhanced data visualization tools.

From the 2019 discussions with U.S. EPA regions and state, local, and Tribal air monitoring agencies, U.S. EPA learned that organizations were in various phases of air sensor and air sensor data adoption. Thus, their air sensor data analysis needs and familiarity with commercially available sensors were highly variable. Some agency needs were focused on understanding the performance of air sensors in different locations, while others wanted to use sensor data to understand local air quality conditions. The available data sources and popular sensor types were variable by organization.

These conversations made it immediately clear that the first step of combining data sources was time-consuming and laborious, and often delayed or impeded the more substantive analyses the agencies wished to do. With limited staff time and technical programming knowledge, agencies repeatedly requested data tools that supported efforts to combine various sources of air pollution and meteorological data that could be used by non-programmers, supported offline data files present on the user’s computer, and included data screening protocols, including data flagging and outlier detection. Agencies also requested tools that supported sensor evaluation, geospatial exploratory analysis, and air pollution and meteorology analyses.

U.S. EPA is not the only organization that has identified these needs in the sensor community. In a recent review article on open air quality data platforms [4], future recommendations include “Simple and accessible tools are needed to bring together multiple sources of information and easily make appropriate comparisons between them. Co-development of these tools between subject matter experts and data users is essential to ensure they provide trustworthy, actionable information.”

There are various existing tools to help analyze air quality and air sensor data using different software platforms and requiring different levels of user expertise. U.S. EPA’s Excel-based macro Analysis Tool allows users to compare air sensor data to air monitor data [22]. U.S. EPA’s Real-Time Geospatial Viewer (RETIGO) is a web-based application that allows users to map spatial air sensor data alongside data from other sources [23]. PLUME Dashboard is an open-source Python-based dashboard for mobile air quality data [24]. The AirSensor R package v1.0 allows users to visualize and understand local air quality using air sensors [25,26]. The Dataviewer application incorporates the functionality of the AirSensor R package into a web application that allows for data to be used by community organizations and citizen scientists [25,26]. Openair is an R package that allows users to import, manipulate, and perform a variety of analyses to understand air pollution data [27]. aiRe is a web-based R-Shiny application that allows users to load, clean, and explore air quality datasets and is designed for the needs of Colombian environmental authorities [28]. Sentinel is an R Shiny app to process and analyze sensor data at the fence line near industrial facilities for pollutants like volatile organic compounds or methane [29]. Aqpet is an R package for air quality policy evaluation [30]. Sensortoolkit is a Python-based library [31] developed at the U.S. EPA to allow users to generate air sensor performance target reports in the same format as those outlined in the U.S. EPA’s air sensor performance target documents [32–35]. Vayu is a Python-based toolbox with a graphical user interface that allows users to perform a variety of visualizations and statistical analyses on air quality data [36]. Giovanni is a web-based tool leveraging surface observations, model output, and satellite remote sensing products so that users can visualize and analyze air quality data [37]. These tools are designed for a variety of users, including R and Python packages requiring coding expertise, and Excel- and web-based tools for less experienced users.

For many of these tools, variation in sensor data formatting limits their use to only a few sensor types, or users face a significant initial coding hurdle at the data import step. For example, the macro analysis tool requires the user to paste in their data for one sensor monitor pair only, and there are size limitations. For RETIGO, import is limited to data in a specified time format and specified header labels; all sensor data needs to look the same and be in a single file. AirSensor is limited to certain sensor types, and for OpenAir, the import needs to be customized for each data format. Many of these tools could become more useful if sensor data formats were standardized.

Efforts to make sensor data formats more consistent could greatly expand the usefulness of these tools and enable users to gather air quality insights more quickly. Some work is ongoing to establish standard formats for air quality data, including work in Colorado (https://cdphe.colorado.gov/air-quality-data-exchange, last accessed 1 July 2025). However, at the time of this writing, no common format has been widely adopted, and it is likely that there will always be a need for different common formats depending on application objectives.

Ultimately, we decided to address these overall needs with two tools: the Air Sensor Network Analysis Tool (ASNAT) [38] and the Air Sensor Data Unifier (ASDU). This paper describes ASDU, an RShiny-based tool we developed to quickly and efficiently reformat air sensor data through a user-friendly interface. This tool has the added functionality of exporting the streamlined data into a variety of formats for easier import into some of the previously mentioned tools. This paper provides some examples of how it can reformat different types of air sensor data. This tool leverages and improves some previous software [31,39]. It is designed to meet the specific needs of U.S. state, local, and tribal air agencies, and U.S. EPA regional staff in efficiently reformatting air sensor data so that it can be used to understand air sensor performance and localized air pollution.

## 2. Materials and Methods

### 2.1. Development

Through our experience working with a variety of air sensors, we realized that air sensor data comes in a variety of data formats. Some are saved as comma-separated values, tab-separated values, or plain text files. Some include metadata, and others do not. Some have header rows that describe the data well; others are missing information (e.g., units) or are missing headers altogether. Timestamps are provided in a number of formats, time zones, and 1–2 column formats. Although sensors of the same make and model often have similar data formats, this is not always the case. These variations were considered in the conceptualization of this tool.

Input was solicited from U.S. EPA Regional staff and state, local, and Tribal agency staff to catalog their needs around air sensor data use. Staff shared their frustrations, most time-consuming tasks, needs, and information about sensors being used in their jurisdictions, and provided sample data for tool testing and design. It was clear that significant time and effort were being spent on reformatting data and that insights could be achieved more quickly if this burden could be reduced.

To provide that support, we developed the Air Sensor Data Unifier (ASDU, an RShiny-based tool [39,40] to allow users to more easily reformat sensor data into standard formats. ASDU includes a dataset dashboard, format wizard, data check, data flagging, and data export functionality. ASDU uses some similar logic to the sensortoolkit Python library [31], but uses a streamlined user interface with interactive options through RShiny. The code is written in R and leverages several existing packages, including shiny [39], shinyjs [41], bslib [42], and DT [43].

### 2.2. Evaluation

The performance of ASDU was briefly evaluated. One test user ran a few example datasets through ASDU and recorded the time it took for a user to go through all steps from running the app to saving the file for ASNAT in all three averaging intervals. Memory usage was recorded using the garbage collection (gc()) function, where the sum of the max used memory for both fixed-sized R objects (Ncells) and variable-sized R objects (Vcells) was recorded after each example dataset.

## 3. Results

### 3.1. Specific User Needs

To begin, we asked U.S. EPA research and program offices, U.S. EPA regions, and state, local, and Tribal air monitoring agency staff to submit a list of air sensors commonly used in their projects or geographical locations. Example data files were compiled for tool development and testing. These files have a variety of header and metadata formats (Table 1) and a variety of naming conventions for the data included (Table S1).

After discussion of the priority functionality of this tool, the txt and csv file types were prioritized. PurpleAir data is different from many other sensor types since most models have duplicate PM_2.5_ measurements (i.e., two Plantower sensors). Raw data from the PurpleAir public API is brought into the U.S. EPA’s RSIG database, and a processed version similar to that available on the U.S. EPA’s fire and smoke map is accessible through ASNAT (i.e., exclude duplicate measurements that disagree, average, and apply US-wide correction) [44]. We decided to load raw PurpleAir csv data directly in ASNAT instead of through ASDU so that the methods would match exactly. Users can use ASNAT to process and export PurpleAir SD card files instead of ASDU.

Some additional instrumentation in use by U.S. EPA and partners was de-prioritized primarily because they were conventional air monitors, higher cost research equipment (e.g., Met One Beta Attenuation Monitor (BAM) (Grants Pass, OR, USA), Environmental-BAM (E-BAM), Aethalometer, Teledyne monitors (Thousand Oaks, CA, USA)), and/or had much more complex measurement output (e.g., metals including Xact 652i cooper environmental, chemical speciation, scanning mobility particle size and condensation particle counter). Although not used specifically for designing the functionality of these tools, some of these types of data may still be able to be reformatted with ASDU.

Some U.S. EPA and external users were interested in direct application programming interface (API) import from a variety of sources, including AirNow, the U.S. EPA’s air quality system (AQS), PurpleAir (all columns), Clarity, Quant AQ, Aeroqual, and AQMesh. After discussing the priorities of the project, the team decided to focus on locally saved data in text formats since most APIs allow users to download data locally, and users could then upload it into ASDU for formatting.

### 3.2. Overall Functionality

**Figure 1.**
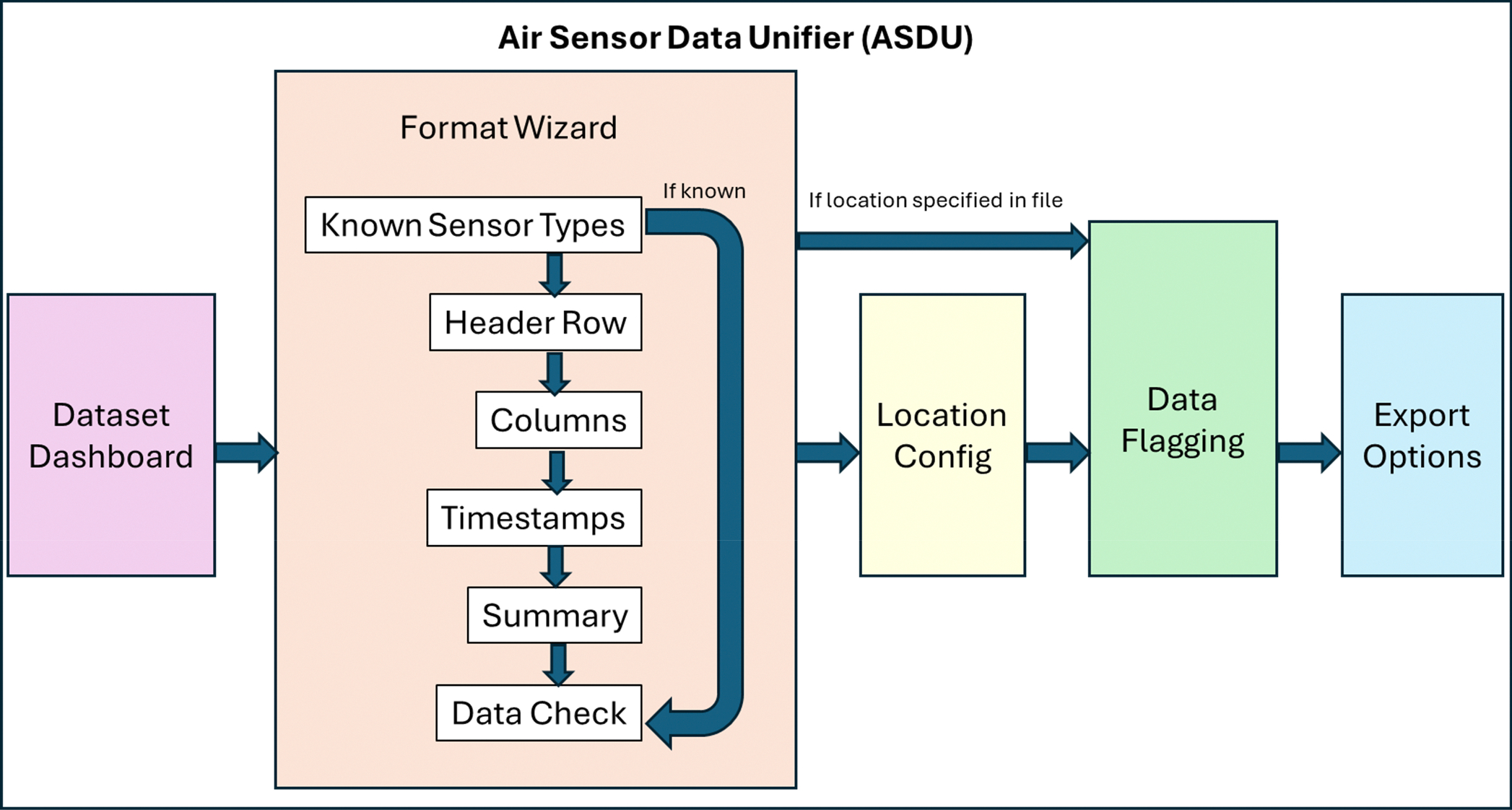
Workflow through the Air Sensor Data Unifier.

Based on the needs and priorities identified, ASDU was developed with the functionality outlined in the following sections. Users load files from their local computers into the data dashboard and then proceed through the steps until they save a local copy of the file(s) in their specified formats (Figure 1).

### 3.3. Dataset Dashboard

**Figure 2.**
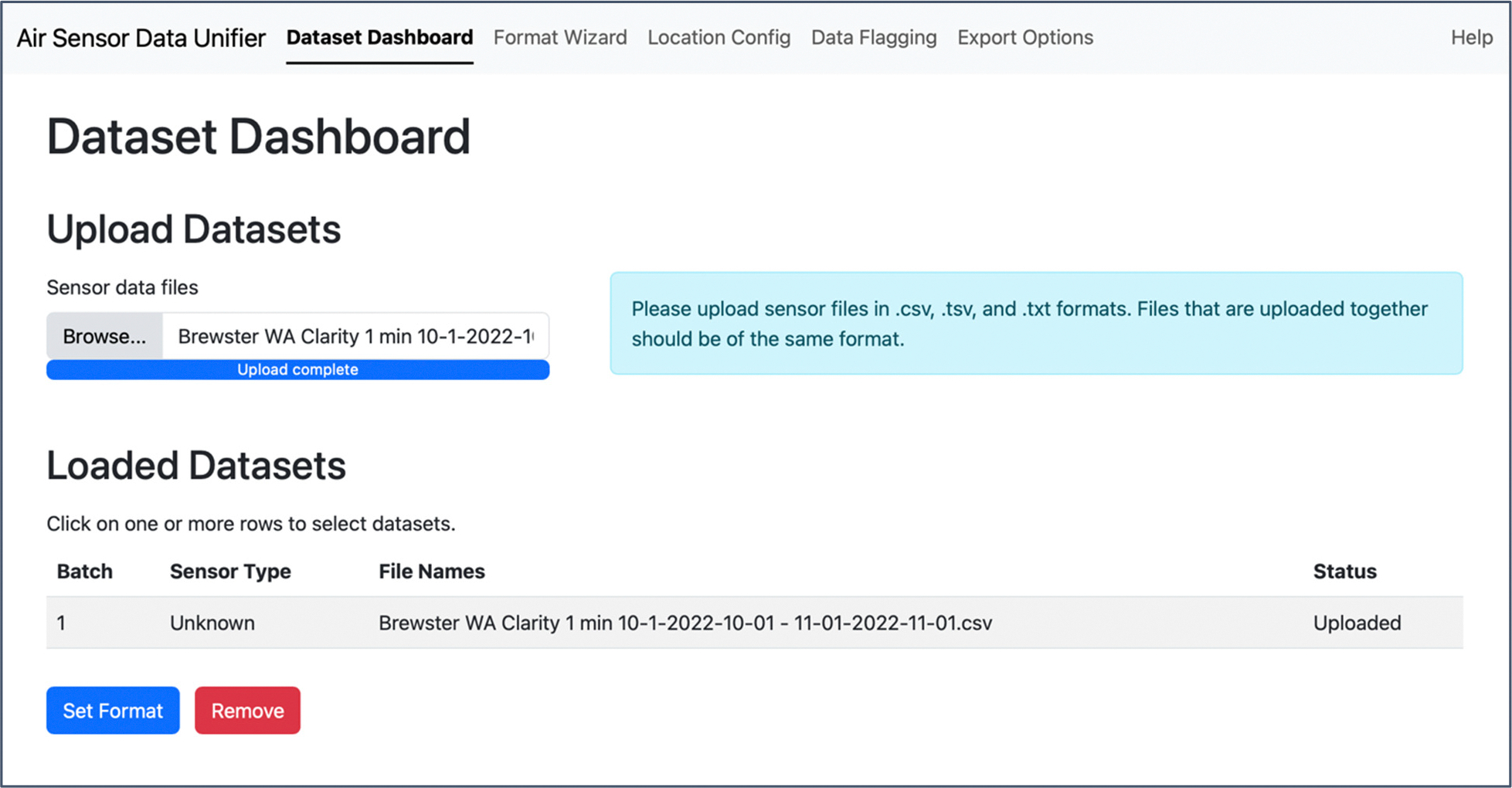
Dataset Dashboard, the first step of the Air Sensor Data Unifier, where batches of air sensor data can be loaded.

Launching the tool opens the Dataset Dashboard. It allows users to upload raw air sensor data files and displays a summary of files already loaded (Figure 2). The browse button can be used to navigate to comma-separated values, tab-separated values, and plain text data files (i.e., file extensions .csv, .tsv, and .txt) located on the user’s computer. If the user has .xlsx files, the user must first open the file in a spreadsheet program (e.g., Microsoft Excel, Google Sheets) and save the file as a .csv or another acceptable file type before loading it into ASDU. Files that are uploaded together should be of the same format. The Air Sensor Data Unifier will check that all the file extensions for a batch are the same. Each dataset’s status is tracked across the application.

### 3.4. Format Wizard

**Figure 3.**
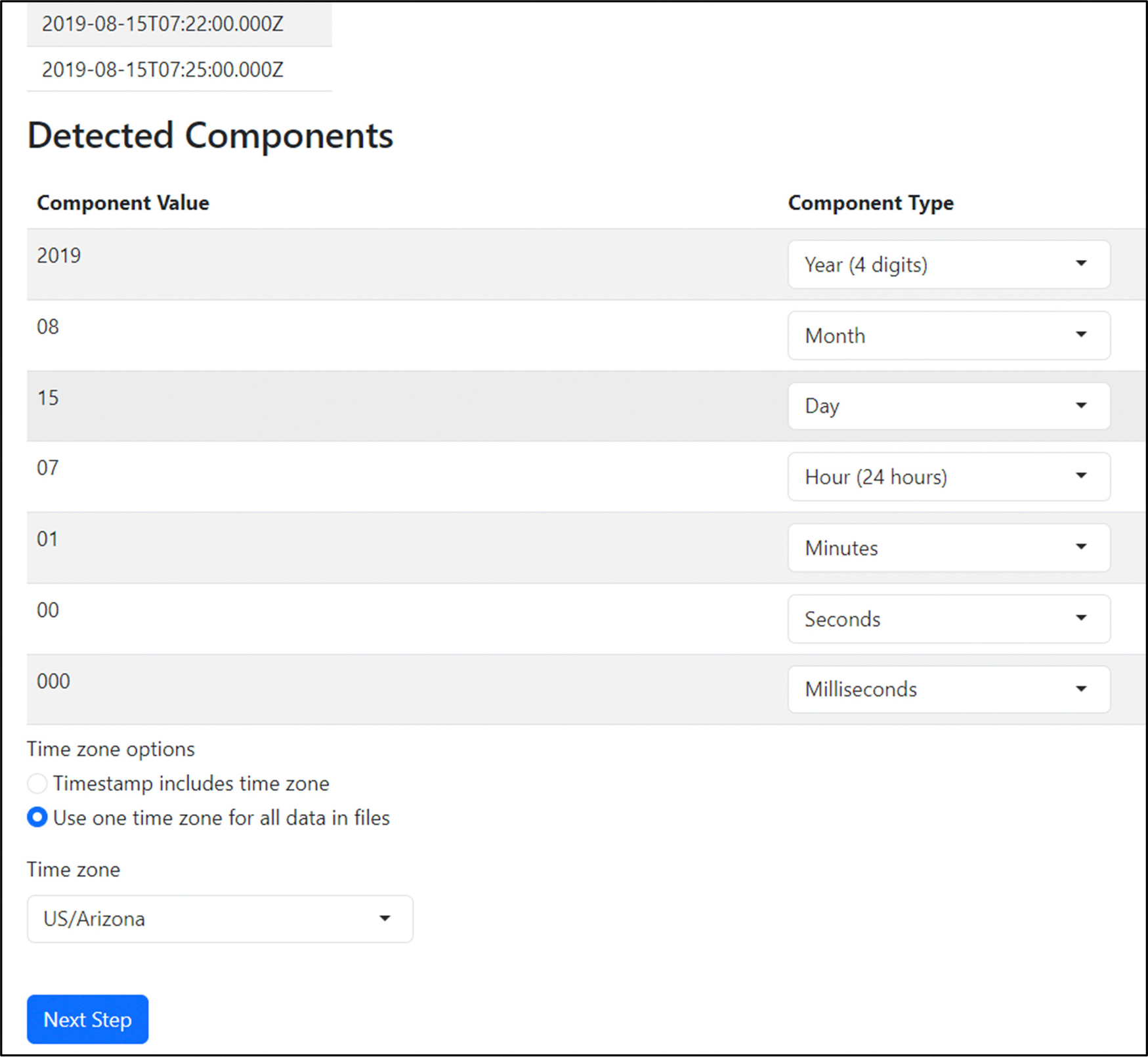
Format Wizard timestamp formatting and time zone options. Timestamp component type is auto-populated, and users can update from the drop-downs as needed.

The Format Wizard tab, found across the top of the screen, allows users to describe the format of their sensor data files. The first (optional) step is to define the data header row. Sensor data comes in many formats; the first row may contain column headers, and the first few rows may contain a variety of metadata, or there may be no column headers. The Format Wizard displays the first 10 lines of the file, and users can specify the header row and the column delimiter for the files. If the header row is not found in the first 10 rows, a button allows you to view additional rows. In the next subtab called Columns, the user can identify the data type and units for the data in each column. Current accepted units include micrograms per cubic meter (μg/m^3^) for particulate matter concentration, parts per billion (ppb) and parts per million (ppm) for gas concentration, number of particles per cubic centimeter (#/cm^3^) and number of particles per cubic meter (#/m^3^) for particle count concentration, degrees Fahrenheit (°F) and degrees Celsius (°C) for temperature, percent (%) for relative humidity, hectopascal (hPa) and pascals (Pa) for pressure, meters per second (m/s) and miles per hour (mph) for windspeed, and degrees for latitude and longitude. “N/A” can be used to indicate that a unit is not applicable. The timestamp format can be further described within the Timestamps subtab. When setting up a new sensor format, the Air Sensor Data Unifier will try to detect the components of any timestamp column(s), and the user can adjust them as needed (Figure 3). The user can also specify the time zone. Finally, the user can save the format information as a JavaScript Object Notation (JSON) file within the Summary subtab. This file can be loaded in subsequent, future runs with data of the same format, so the user does not have to re-specify the format information.

Lastly, the Data Check subtab scans the full data file and provides the starting and ending timestamps. Plots of the first 10 sensor data values are also shown. This can give users an idea of whether they have loaded and described the data as anticipated.

**Figure 4.**
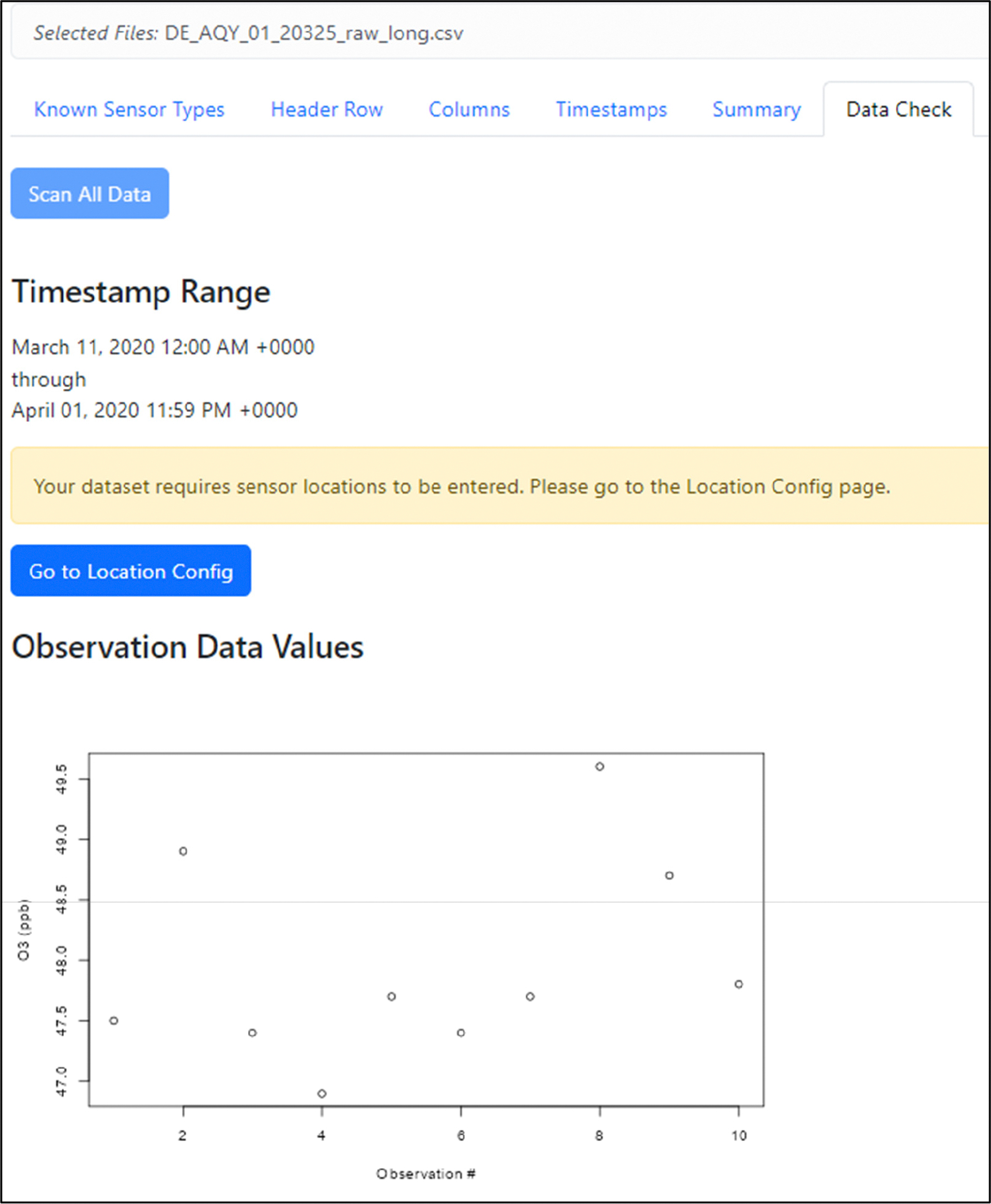
Sensor data check showing the timestamp range and the values of the first ten observations plotted.

Figure 4 shows that the file loaded for this example spanned from March 11 until April 1, 2020. If this is not the time period the user expected to load, they could go back and modify the data they loaded before proceeding and/or double-check their descriptions of the timestamp. The plot shows the first few values of ozone (O_3_) data from this example file. This can help the user ensure the values are reasonable and there is no misalignment in the column that was selected. However, it is important to note that many air sensors have bias, influence from environmental conditions, or interference from other pollutants, so these values may need further correction to be more comparable to true concentration values.

### 3.5. Location Config

Latitude and longitude may be specified within the data file or recorded separately. The user can add the location for each sensor within the Location Config main level tab across the top of the screen. A single sensor ID may have multiple locations listed in the file, but only one location can be specified in the location configuration tab. This means that mobile data can be loaded through ASDU, as long as the latitude and longitude are included as columns in the data file.

### 3.6. Data Flagging

**Figure 5.**
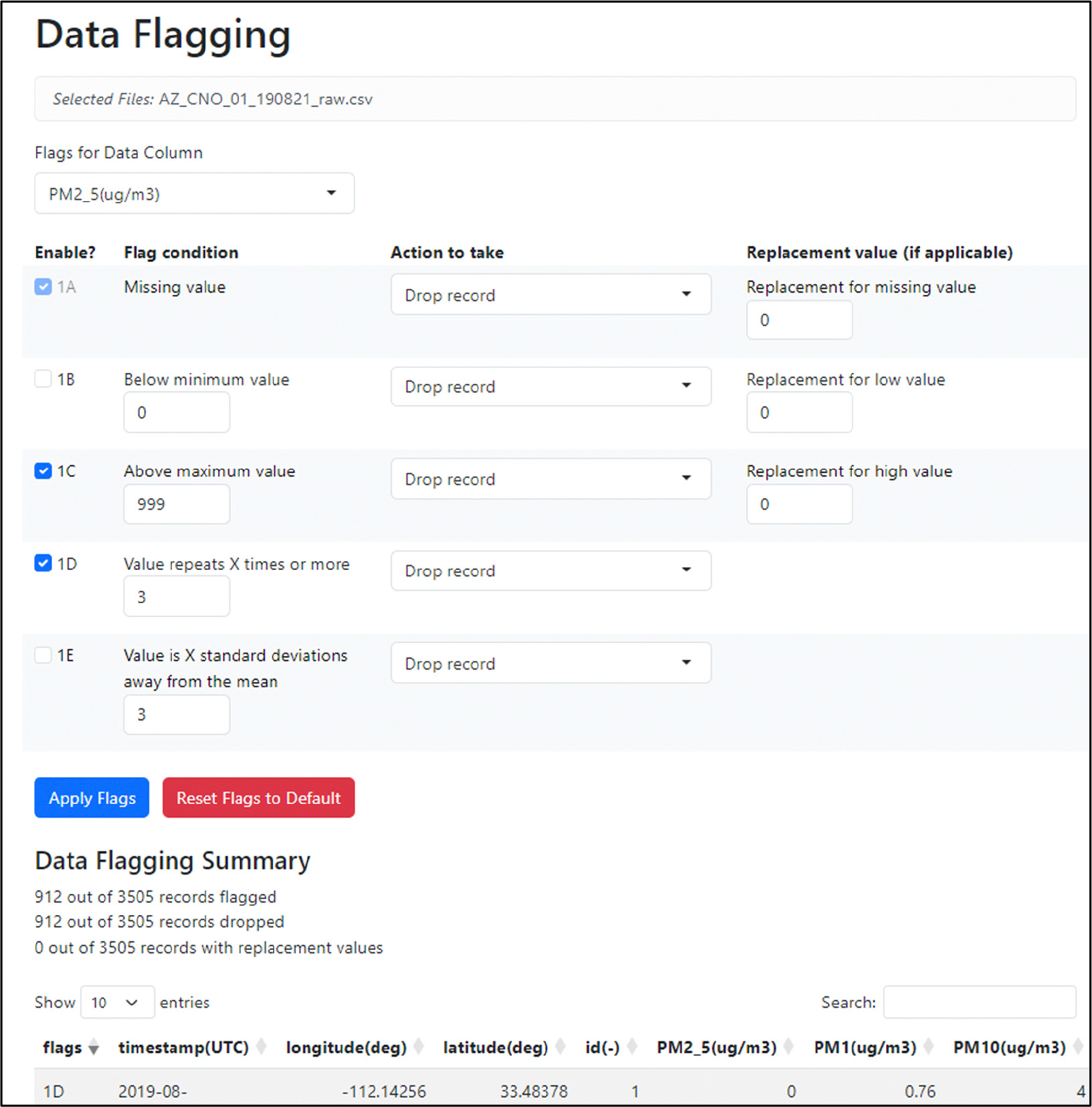
Data Flagging functionality, including the ability to apply flags and then see how much data and which points will be removed.

The Data Flagging tab allows users to set up rules for checking the data and specify how those conditions should be handled (Figure 5). Flags can be set up for each data column in the dataset. There are five data flags that can be applied: (1) handling of a missing value, (2) below minimum value, (3) above maximum value, (4) repeated value for a user-specified number of data points, and (5) outlier value by user-specified number of standard deviations away from the mean. Each flag has an identifier based on the data column’s index (starting with 1) and the flag (letters A through E). This identifier is reported in a new “flags” column when the flags are applied to the dataset to identify which flags matched each record. Missing values are always dropped, and timestamps without data are not saved in the final file. The Data Flagging Summary will list how many records from the dataset were flagged, how many records will be dropped when the data is exported, and how many records will have replacement values when exported. The user can export the dataset with or without the flagged data.

In this example (Figure 5), missing values are excluded, high outliers (>999 μg/m^3^) are excluded, and values that repeat more than three times are excluded. This leads to 26% of the data being excluded. This would be an example where the user may want to open their data file and further explore any issues before proceeding, since 26% is a large amount of data to exclude. While it is unlikely that true PM_2.5_ concentrations are stable in an area for 3 h, in this example, there are some periods of repeat zeros, likely due to concentrations being below the sensor’s limit of detection. Depending on the objectives of the analysis, the user may not want to remove the repeat zeros from the analysis, as it may bias their hourly or 24-h averages high, and the user would want to uncheck this option before proceeding to export their averaged dataset. However, it is important to understand typical failure patterns of the sensor used, as repeat zeros can indicate a failure, as opposed to concentrations below the detection limit for some sensor types [45].

Users who take the data set generated in the ASDU and use it in the Air Sensor Network Analysis Tool (ASNAT) will have another opportunity to remove outliers from the dataset in ASNAT. However, some issues may be clearer in the recorded time resolution but may be obscured at the longer averages (e.g., minute spikes to a very high value indicating sensor blockage). In some cases, users may identify issues in ASNAT and realize that it would be helpful to go back and adjust the flagging in the recorded resolution data in ASDU before averaging again.

### 3.7. Export Options

The Export Options tab allows the user to select how they want the data to be exported and will create the reformatted data files. ASDU can export data in the following formats: the ASNAT Standard Format File, Keyhole Markup Language (KML) (for use in Google Earth or Geographic Information System (GIS) programs), and in the format used by RETIGO (https://www.epa.gov/hesc/real-time-geospatial-data-viewer-retigo, last accessed 20 February 2025). Data averaging can also be applied to the file. The current output options are “raw”, where no averaging is performed, “hourly”, or “daily” (currently 24-h averages in coordinated universal time (UTC)).

### 3.8. Feedback and Improvements So Far

Before public release on GitHub, the tool was beta-tested by several U.S. EPA and external users in the fall of 2024. Feedback was summarized and addressed before public release in Spring 2025, and, since public release, additional feedback has come in and been addressed. Most feedback on ASDU was provided by staff at three state and local agencies from different regions of the U.S. This feedback is summarized in Table 2. Although most of the feedback has been addressed, remaining concerns will be prioritized in future updates, which are dependent on the resources available for the project. Testing and feedback from partners have been incredibly valuable, as many agencies tested using data file formats that were not included in our original development dataset (e.g., QuantAQ datasets) or with data formats that have been updated by the manufacturer since the time of testing. In addition, they highlighted key features that we were not aware were priorities or did not consider in the original development.

### 3.9. Performance Examples

We ran several example sensor datasets through ASDU, which were subsets of data from our long-term performance project [45]. It took 4–8 min to reformat and save different sensor data types, with less time (≤4 min) to load data of known formats where an existing formatting json was used (Table 3). These times are approximate as user speeds will vary depending on the user’s experience with the tool, whether they have all the needed information quickly accessible (e.g., latitude and longitude, units on variables), any mistakes are made, and how many variables they want to identify in the dataset. Users could spend more time flagging the data. Times to run would not be largely different if users were exporting data into the other export formats (e.g., KML, RETIGO), but could be faster if only one file type were exported.

## 4. Discussion

This work has resulted in a user-friendly, RShiny-based, sensor data reformatting tool capable of reducing the considerable burden of harmonizing data formats. This work enables national, state, local, and Tribal air agencies, consultants, academics, and others to quickly and efficiently combine data from several sensor networks so that the data can be used for sensor performance testing, air quality analysis, and decision making.

While the tool has been designed to be relatively sensor-agnostic, there are still some formatting requirements. The tool currently works with datasets where there is one row of data (any number of columns) per timestamp. In addition, data must be associated by column (e.g., PM_2.5_ data in column X) and be saved as .txt, .csv, or .tsv file types. The data must include a timestamp column and at least one observation column for ozone (O_3_), nitrogen dioxide (NO_2_), carbon monoxide (CO), particulate matter (PM), particle count, or meteorology data. The size of data that can be loaded and processed at the same time is also limited by local computing resources. Some of these limitations may be improved in later versions of this tool, depending on the priorities of users and the availability of funding.

Existing air sensor and air quality data tools are often focused on a single manufacturer’s sensor [25,26] and may require coding experience [25,27,30,46] or data in a specific format [23,25,28]. ASDU allows users to load data from a wide variety of sensor types, does not require coding experience, and has very limited requirements for a specific input format. In addition, ASDU allows users to load data from a local download and does not require the data to be online, reported to the cloud, or publicly available. This functionality may be especially important in areas without Wi-Fi or cellular reception and in areas where data is sensitive and groups prefer to keep data private and only share once the results are finalized.

A variety of different data formatting conventions and standards exist for air quality data and metadata. It is challenging to identify a universal data format since all formats have strengths and limitations and may be more or less ideal for different applications. For example, some formats are better for efficient data transmission, while others may minimize data storage size requirements. With so many different common formats, a tool like the air sensor data unifier is essential to help efficiently move data from one format to another. Interoperability is critical as many users may be interested in using air sensor data in multiple different tools simultaneously to achieve their data analysis goals and produce project results.

Past research has shown the value of R-Shiny-based tools as they allow diverse users to efficiently employ complex methodologies without requiring extensive training [47]. More than two hundred air quality professionals have attended training on this tool. Training a new user takes approximately 30 min, which is significantly shorter than the time required to train staff in reformatting data using software like R (v 4.5.1) or Python (v 3.13.7), especially for those without prior experience. Dedicated office hours have allowed users to discuss roadblocks and obtain support. Users currently include staff from air agencies (such as state, local, Tribal, and U.S. EPA), other federal agencies, academia, consulting firms, and various other organizations.

One of the challenges in developing air quality data tools is targeting the right user base. As illustrated in our air sensor data dialog discussions [20,21], different state and local agencies have different capacities and levels of expertise. Some local agencies feel that they are not the target audience for a tool like ASDU since they have so much in-house data analysis expertise, but feel that it is a tool for them to pass on to the community groups they work with. Community groups also have a wide range of capacities and desires. Some groups desire professional-level reports prepared by technical experts (e.g., contract staff), while other groups are focused on education (e.g., middle school groups) and are not interested in the data beyond colors and screen outputs, and there are many groups that fall somewhere in between, where data tools like ASDU may help. Our dialogs also found that there may be some regional differences in the engagement with air sensors (e.g., from East Coast and West Coast agencies). Some West Coast agencies have advanced sensor programs, including custom-built state sensor networks (e.g., Oregon, Washington), sensor evaluation programs (e.g., South Coast Air Quality Management District Air Quality Sensor Performance Evaluation Center), and custom-built air sensor software (e.g., South Coast Air Quality Management District, Puget Sound Clean Air Agency). These agencies with extensive technical staff may be more likely to use custom data analysis to accomplish agency or community agency partnership goals. However, these agencies can still benefit from these tools either by using them directly or by using pieces of the open-source code that may be helpful for their analyses. Some regional differences may be due to increased knowledge and engagement of the public after wildfires [48]. Continued work is needed to advertise these tools to suitable audiences, update the tools to better meet real-world needs, and provide tailored training to varied audiences.

So far, users have reported using this tool to quickly reformat data from a short-term city-wide network to further explore the dataset in ASNAT. This tool streamlines the analysis pipeline, allowing for similar data formatting steps to be quickly repeated. The simplicity of the R-Shiny interface can allow for less disruption when staffing changes occur, requiring new staff to be trained to take over the analysis.

With the wide variety of users trained and engaged with this tool, a number of suggested improvements have already come in, and the tool has been improved since its initial release. This work is ongoing, and we hope to continue improving the functionality based on the feedback from initial users. In addition, the code is publicly available so that others may modify it for their specific use cases and/or integrate it into existing data analysis tools as needed.

This tool contributes to increased accessibility of air quality monitoring data. Reducing the time needed to reformat air sensor data reduces the barrier to entry and allows more users to use air sensors for a variety of uses. This can allow for further democratization of air quality monitoring. In addition, this saves resources that can be put into further analysis, data communication, or other critical programs. While our development was mostly focused on U.S. EPA stakeholder needs (e.g., state, local, and Tribal agencies), there are a number of other groups who may benefit from this kind of application. This may include researchers, including undergraduate researchers or course-based undergraduate research experiences, who may be interested in air quality data but lack the technical expertise to deal with the import process. Air quality data is not only of interest to air quality researchers but also to public health scientists, urban planners, and ecologists, and these researchers also may be able to use the tool to streamline the data as a first step to gaining valuable insights into environmental and health-related impacts.

Much has been learned about air quality and air pollution in recent decades. However, many questions remain, especially at local and individual levels and in low-resource settings, where air quality data and information about air pollution have been less available. More efficient processing of air sensor data allows for more time to be spent digging into pressing public health questions. In addition, expanded access to air quality sensors and data can generate greater motivation and awareness of air pollution [5,49]. The implications of air sensor use to improve public health are an area of ongoing research [50].

## 5. Conclusions

The development and public release of ASDU represent a significant advancement in the management and use of air sensor data. By addressing the challenges of data format variability and providing a streamlined, user-friendly interface for data processing, ASDU empowers air quality professionals to efficiently integrate sensor data into their workflows. This tool not only facilitates basic quality control and data export into standard formats but also enhances the interoperability of air sensor data across various applications, including ASNAT, Google Earth, and GIS programs. It decreases the amount of time and energy spent on data wrangling and allows non-programmers to assist with this task and users to spend more project time gaining insights from the data.

By understanding the diverse needs and constraints faced by U.S. EPA, state, local, and Tribal air quality organizations, ASDU is designed to meet their specific requirements, enabling more effective air quality analysis. The tool’s adaptability to various sensor types and its open-source nature further democratize the use of air quality sensors, providing broader access to high-resolution air quality data.

Looking forward, continued collaboration with air quality professionals and stakeholders will be essential to refine ASDU’s functionalities and expand its capabilities. Since the tool is open source and available on GitHub, external users can improve and make public their own versions of the code set. By fostering an environment of shared learning and innovation, the ASDU development team aims to contribute to the ongoing efforts to improve air quality monitoring and public health outcomes. As air sensor technology continues to evolve, tools like ASDU will play a pivotal role in leveraging these advancements to address complex environmental challenges.

## Supplementary Material

Supplement1

**Supplementary Materials:** The following supporting information can be downloaded at https://www.mdpi.com/article/10.3390/air3030021/s1, Table S1: Priority datasets for inclusion into ASDU headers for each file.

## Figures and Tables

**Table 1.** Priority datasets for inclusion into ASDU.

Manufacturer	Model	File Format	Header and Meta Data Format
Aeroqual (Auckland, New Zealand)	AQY	csv	5 rows of metadata, Row 7 header
Aeroqual (Auckland, New Zealand)	AQY-R	csv	Same as AQY
Airly Inc. (Palo Alto, CA, USA)	Airly	csv	Row 1 header
APIS (Grants Pass, OR, USA)	APIS	csv	Row 1 header
Applied Particle Technology (Boise, ID, USA)	Maxima	csv	Row 1 ID Row 2 headers
Clarity Movement Co. (Berkeley, CA, USA)	Node-S	csv	Row 1 header
Davis Instruments (Hayward, CA, USA)	AirLink	xlsx	Row 1 header including ID and location in each description
Dylos corporation (Riverside, CA, USA)	Dylos	txt	Row 1 header
Ecomeasure (Saclay, France)	Ecomeasure_SGS	xlsx	Row 1–3 metadata, Row 5 header
Habitat Map (Brooklyn, NY, USA)	AirBeam1	csv	Row 1–3 header
Habitat Map (Brooklyn, NY, USA)	AirBeam2	csv	Rows 1–9 header data
Habitat Map (Brooklyn, NY, USA)	AirBeam3	csv	Row 1–9 header
IQAir (Goldach, Switzerland)	AirVisual Pro	csv	Row 1 header
Kunak (Navarra, Spain)	Air Pro	csv	Row 1 metadata Row 2 header
Myriad Sensors (Brentwood, TN, USA)	Pocket Lab Air	csv	Row 1 header
PurpleAir (Draper, UT, USA)	PA-II-SD	csv	Row 1 header
Sensirion (Stäfa, Switzerland)	SEN44	xlsx	Rows 1–11 metadata, Rows 12, 13 headers
Sensit Technologies (Valparaiso, IN, USA)	RAMP	txt	No header, variable ID included in column before value
TSI (Shoreview, MN, USA)	BlueSky	csv	Row 1 header, Row 2 units
WA Department of Ecology (Lacey, WA, USA)	Custom-built with Sensiron ^1^	csv	No headers

1 Downloaded through Envista ARM, DR DAS, Granville, OH, USA.

**Table 2.** User feedback and updates to ASDU.

Feedback	Reason	Version	Addressed
Better timezone handling	Although daylight savings time is not preferred for most air monitoring applications, some data may still come in daylight time and need adjustment	Beta test version	Yes
Better time format detection and error handling	Some example datasets were not correctly loaded	Beta test version	Yes
Consider more than 10 header rows	Some datasets have many rows before the header	Beta test version	User can now advance through subsequent rows
Improved error handling on latitude and longitude	Backwards latitude and longitude crashes ASNAT	Beta test version	Yes
Better documentation needed on averaging method		Beta test version	Added documentation (e.g., 11:00 to 11:59 labeled as 11:00)
Add pressure data type		Beta test version	Yes
Allow user to remove problematic data		Beta test version	Data flagging added
Data rounding	Too many decimal places included in the sensor data. Not enough decimal places included in the latitude and longitude.	Beta test version, Public version	Yes
Allow larger file uploads	High-time resolution data (e.g., minutes) can generate large files quickly	Public version	100 MB max file size
Improve installation error	Library version conflict	Public version	Yes
Assign unique sensor IDs if location changes	Sensors may be stationary but rotate through multiple sites for quality assurance or other reasons throughout a project	Public version	Yes
Ensure output data is sorted by timestamp and sensor ID	Needed if multiple sensors are then loaded to ASNAT	Public version	Yes
Sensor API direct import (e.g., Clarity, QuantAQ)	Save users the step from API download, then ASDU upload.	Public version	Potential future priority
Have a publicly hosted tool	Save users from needing to install R and dependent libraries	Beta test version, Public version	Potential future priority
Allow user to create custom Data Types, Extensions, and Units		Beta test version	Potential future priority

**Table 3.** Example performance benchmarks.

Example	Rows Raw Data	Sensors	Days	Max Used Memory (Mb)	Time for User to Run (Unknown Format)	Time for User to Run (Known Format)

Aeroqual AQY	34,126	1	22	407.6	3 min, 55 s	1 min, 31 s
APT Maxima	158,208	1	56	513.4	4 min, 44 s	1 min, 58 s
Clarity Node	86,542	3	331	271.7	8 min, 4 s	3 min, 33 s

## Data Availability

The code is available on GitHub https://github.com/USEPA/air-sensor-data-unifier (accessed on 26 August 2025).

## Abbreviations

The following abbreviations and acronyms are used in this manuscript:

AI: Artificial Intelligence
API: Application Programming Interface
AQS: Air Quality System
ASDU: Air Sensor Data Unifier
ASNAT: Air Sensor Network Analysis Tool
BAM: Beta Attenuation Monitor
°C: Degrees Celsius
CO: Carbon monoxide
Csv: Comma-separated values
E-BAM: Environmental-Beta Attenuation Monitor
EPA: Environmental Protection Agency
°F: Degrees Fahrenheit
GIS: Geographic Information System
hPa: Hectopascal
JSON: JavaScript Object Notation
KML: Keyhole Markup Language
m/s: Meters per second
mph: Miles per hour
N/A: Not applicable
NO_2_: Nitrogen dioxide
O_3_: Ozone
ORD: Office of Research and Development
Pa: Pascals
PM: Particulate matter
Ppb: Parts per billion
Ppm: Parts per million
RETIGO: Real Time Geospatial Data Viewer
tsv: Tab-separated values
txt: Plain text files
UNC: University of North Carolina at Chapel Hill
U.S.: United States
UTC: Coordinated universal time
Wi-Fi: Wireless fidelity
μg/m^3^: Micrograms per cubic meter
#/cm^3^: Number of particles per cubic centimeter
#/m^3^: Number of particles per cubic meter
