# Supplementary material for: Air Sensor Data Unifier: R-Shiny Application
Source: Air (Basel). Author manuscript; Available in PMC 2026 Aug 30. (PMC12510237; doi:10.3390/air3030021)
Supplement: Supplementary file 1 [file NIHMS2114021-supplement-Supplement1.zip › air-3797056-supplementary.pdf]

Supplemental Information: Air Sensor Data Unifier: R-Shiny application

Karoline K. Barkjohn[1], Catherine Seppanen[2], Saravanan Arunachalam[2], Stephen Krabbe[3], Andrea L. Clements[1]

[1] United States Environmental Protection Agency Office of Research and Development, United States of America; Barkjohn.karoline@epa.gov, Clements.Andrea@epa.gov
[2] Institute for the Environment, The University of North Carolina at Chapel Hill, United States of America; cseppan@email.unc.edu, sarav@email.unc.edu
[3] United States Environmental Protection Agency Region 7, United States of America; krabbe.stephen@epa.gov
* Correspondence: Barkjohn.karoline@epa.gov

Table S1. Priority datasets for inclusion into ASDU headers for each file.

Manufacturer	Model	Header format	Data included in columns
Aeroqual (Auckland, New Zealand)	AQY	5 rows of meta data (ID, time period, time zone, averaging period), Row 7 header	Time<br>NO2 (ppb)<br>O3 (ppb)<br>PM2.5 (μg/m³)<br>TEMP (°C)<br>RH (%)<br>DP (°C)<br>Inlet<br>Journal category<br>Created by<br>Details
Aeroqual (Auckland, New Zealand)	AQY-R	Same as AQY	Same as AQY without last 3 data columns
Airly Inc. (Palo Alto, CA, U.S.)	Airly	Row 1 header	installation_<br>id<br>date<br>temperature<br>humidity<br>pressure<br>pm10<br>pm25<br>pm1<br>no2<br>o3
APIS (Grants Pass, OR, U.S.)	APIS	Row 1 header	timestamps<br>CO<br>NO<br>NO2<br>NOx<br>O3<br>baro

			dew<br>rh<br>temp
Applied Particle Technology (Boise, ID, U.S.)	Maxima	Row 1 ID<br>Row 2 headers	Timestamp<br>Temperature(C)<br>Humidity(%)<br>Pressure(Pa)<br>Counts_0.3um(#/0.1L)<br>Counts_0.5um(#/0.1L)<br>Counts_1.0um(#/0.1L)<br>Counts_2.5um(#/0.1L)<br>Counts_5.0um(#/0.1L)<br>Counts_10.0um(#/0.1L)<br>PM1.0(ug/m3)<br>PM2.5(ug/m3)<br>PM10(ug/m3)<br>PM1_Atm(ug/m3)<br>PM2.5_Atm(ug/m3)<br>PM10_Atm(ug/m3)
Clarity Movement Co. (Berkeley, CA, U.S.)	Node-S	Row 1 header	nodeId<br>timeUtc<br>aqi<br>pm1MassConcCalib[ug/m3]<br>pm1MassConcRaw[ug/m3]<br>pm2_5MassConcCalib[ug/m3]<br>pm2_5MassConcRaw[ug/m3]<br>[pm10MassConcCalib[ug/m3]<br>pm10MassConcRaw[ug/m3]<br>no2ConcCalib[ppb]<br>no2ConcRaw[ppb]<br>pmCoarseMassConcRaw[ug/m3]<br>so2ConcRaw[ppb]<br>o3ConcRaw[ppb]<br>coConcRaw[ppb]<br>noConcRaw[ppb]<br>noxConcRaw[ppb]<br>pressure[kPa]<br>dewPoint[degC]<br>temperature[degC]<br>relHumidity[%]<br>tVocConc[ppb]<br>eCo2Conc[ppb]<br>latitude<br>longitude
Davis Instruments	AirLink	Row 1 header including ID and location in	DateTime_23_AIRS_raw<br>PM1_23_AIRS_raw<br>PM25_23_AIRS_raw

(Hayward, CA U.S.)		each description	PM10_23_AIRS_raw<br>Temp_23_AIRS_raw<br>RH_23_AIRS_raw
Dylos corporation (Riverside, CA, U.S.)	Dylos	Row 1 header	Date/Time,<br>Small,<br>Large
Ecomeasure (Saclay, France)	Ecomeasure_SGS	Row 1-3 meta data (Serial number, comment, from to date time)	Date (UTC)<br>Date (US/Pacific)<br>SIM Serial Number ( )<br>Temp (deg F)<br>RH (%)<br>Pressure (mbar)<br>CO (ppm)<br>NO2 (ppm)<br>O3 (ppb)<br>PM 1 (ug/m3)<br>PM 2.5 (ug/m3)<br>PM 10 (ug/m3)<br>CO inst (ppm)<br>NO2 inst (ppm)<br>O3 inst (ppb)<br>CO Raw (raw)<br>NO2_Raw (raw)<br>O3 Raw (raw)
Habitat Map (Brooklyn, NY, U.S.)	AirBeam1	Row 1-3 header including the serial number in the headers (in this example 0018961059DD)	sensor:model<br>AirBeam-RH<br>Timestamp<br><br>sensor:package<br>AirBeam:0018961059DD<br>geo:lat<br><br>sensor:capability<br>Humidity<br>geo:long<br><br>sensor:units<br>percent<br>Value
Habitat Map (Brooklyn, NY, U.S.)	AirBeam2	Rows 1-9 header data including the serial number in the headers (in this example 0011E4000546)	Sensor_Package_Name<br>Airbeam2-0011E4000546<br>Sensor_Name<br>AirBeam2-F<br>Measurement_Type<br>Temperature<br>Measurement_Units

			fahrenheit<br>1:Measurement_Value<br><br>Sensor_Package_Name<br>Airbeam2-0011E4000546<br>Sensor_Name<br>AirBeam2-PM1<br>Measurement_Type<br>Particulate Matter<br>Measurement_Units<br>microgram per cubic meter<br>2:Measurement_Value<br><br>Sensor_Package_Name<br>Airbeam2-0011E4000546<br>Sensor_Name<br>AirBeam2-PM10<br>Measurement_Type<br>Particulate Matter<br>Measurement_Units<br>microgram per cubic meter<br>3:Measurement_Value<br><br>Sensor_Package_Name<br>Airbeam2-0011E4000546<br>Sensor_Name<br>AirBeam2-PM2.5<br>Measurement_Type<br>Particulate Matter<br>Measurement_Units<br>microgram per cubic meter<br>4:Measurement_Value<br><br>Sensor_Package_Name<br>Airbeam2-0011E4000546<br>Sensor_Name<br>AirBeam2-RH<br>Measurement_Type<br>Humidity<br>Measurement_Units<br>percent<br>5:Measurement_Value
Habitat Map (Brooklyn, NY, U.S.)	AirBeam3	Row 1-9 Header including the serial number in the headers	ObjectID<br>Session_Name<br>Timestamp<br>Latitude<br>Longitude

		(in this example AirBeam3-e868e738aa60)	Sensor_Package_Name<br>AirBeam3-e868e738aa60<br>Sensor_Name<br>AirBeam3-F<br>Measurement_Type<br>Temperature<br>Measurement_Units<br>fahrenheit<br>1:Measurement_Value<br><br>Sensor_Package_Name<br>AirBeam3-e868e738aa60<br>Sensor_Name<br>AirBeam3-PM1<br>Measurement_Type<br>Particulate Matter<br>Measurement_Units<br>microgram per cubic meter<br>2:Measurement_Value<br><br>Sensor_Package_Name<br>AirBeam3-e868e738aa60<br>Sensor_Name<br>AirBeam3-PM10<br>Measurement_Type<br>Particulate Matter<br>Measurement_Units<br>microgram per cubic meter<br>3:Measurement_Value<br><br>Sensor_Package_Name<br>AirBeam3-e868e738aa60<br>Sensor_Name<br>AirBeam3-PM2.5<br>Measurement_Type<br>Particulate Matter<br>Measurement_Units<br>microgram per cubic meter<br>4:Measurement_Value<br><br>Sensor_Package_Name<br>AirBeam3-e868e738aa60<br>Sensor_Name<br>AirBeam3-RH<br>Measurement_Type<br>Humidity

			Measurement_Units<br>percent<br>5:Measurement_Value
IQAir (Goldach, Switzerland)	AirVisual Pro	Row 1 header	Timezone<br>Datetime<br>AQI US<br>AQI CN<br>PM2.5 (ug/m3)<br>PM10 (ug/m3)<br>CO2 (ppm)<br>Temperature (Celsius)<br>Temperature (Fahrenheit)<br>Humidity (%)<br>HCHO (ppb)<br>TVOC (ppb)
Kunak (Navarra, Spain)	Air Pro	Row 1 “Data Validated + Temporary”<br>Row 2 header	Device<br>Datetime<br>CO GCc (ppb)<br>Flag CO GCc<br>Description CO GCc<br>Humidity ext (%)<br>Flag Humidity ext<br>Description Humidity ext<br>NO2 GCc (ppb)<br>Flag NO2 GCc<br>Description NO2 GCc<br>NO GCc (ppb)<br>Flag NO GCc<br>Description NO GCc<br>NOx GCc (ppb)<br>Flag NOx GCc<br>Description NOx GCc<br>O3 GCc (ppb)<br>Flag O3 GCc<br>Description O3 GCc<br>PM1 (ug/m3)<br>Flag PM1<br>Description PM1<br>PM2.5 (ug/m3)<br>Flag PM2.5<br>Description PM2.5<br>PM10 (ug/m3)<br>Flag PM10<br>Description PM10<br>Temp ext (C)<br>Flag Temp ext<br>Description Temp ext

Myriad Sensors (Brentwood, TN, U.S.)	Pocket Lab Air	Row 1 header	Date<br>Time<br>Elapsed Seconds<br>lat<br>lng<br>Particulate Matter-PM10 (ug/m³)<br>Particulate Matter-PM2.5 (ug/m³)<br>Particulate Matter-PM1.0 (ug/m³)<br>Particulate Matter-10 Min. Avg. PM10 (ug/m³)<br>Particulate Matter-10 Min. Avg. PM2.5 (ug/m³)<br>Particulate Matter-10 Min. Avg. PM1.0 (ug/m³)<br>Ozone-10 Min. Avg. Ozone (ppb)<br>Ozone-Ozone (ppb)<br>Carbon Dioxide-CO₂ (ppm)<br>Carbon Dioxide-10 Min. Avg. CO₂ (ppm)<br>Air Quality Index-AQI Air Quality Index-10 Min. Avg. AQI<br>Measurement Conditions-Temperature (℃)<br>Measurement Conditions-Humidity (%RH)<br>Measurement Conditions-Pressure (mBar)<br>Measurement Conditions-Light Intensity (lux)
PurpleAir (Draper, UT, U.S.)	PA-II-SD	Row 1 header	UTCDateTime<br>mac_address<br>firmware_ver<br>hardware<br>current_temp_f<br>current_humidity<br>current_dewpoint_f<br>pressure<br>adc<br>mem<br>rssi<br>uptime<br>pm1_0_cf_1<br>pm2_5_cf_1<br>pm10_0_cf_1<br>pm1_0_atm<br>pm2_5_atm<br>pm10_0_atm<br>pm2.5_aqi_cf_1<br>pm2.5_aqi_atm<br>p_0_3_um<br>p_0_5_um

			p_1_0_um<br>p_2_5_um<br>p_5_0_um<br>p_10_0_um<br>pm1_0_cf_1_b<br>pm2_5_cf_1_b<br>pm10_0_cf_1_b<br>pm1_0_atm_b<br>pm2_5_atm_b<br>pm10_0_atm_b<br>pm2.5_aqi_cf_1_b<br>pm2.5_aqi_atm_b<br>p_0_3_um_b<br>p_0_5_um_b<br>p_1_0_um_b<br>p_2_5_um_b<br>p_5_0_um_b<br>p_10_0_um_b<br>gas
Sensirion (Stäfa, Switzerland)	SEN44	Rows 1-11 meta data (EdfVersion, date, Application name, application version, file path, host name, measurement group, measurement session, operation system, sensor ID, T offset), Rows 12, 13 column headers including device IDs (SEN44_(1d6f75), 733F37AC73168E94 in this example)	# Type=float64,Format=.1f,Unit=s<br>Epoch_UTC<br><br>Type=string,Format=<br>Local_Date_Time<br><br>Device=SEN44_(1d6f75),Device_Serial_Number=SEN44-733F37AC73168E94,Port=0,SensorName=SEN44_8E94,Sensor_Serial_Number=733F37AC73168E94,Signal=Mass_Concentration_1p0,Type=float,Format=.6f,Unit=ug/m³,Product=SEN44,Measurement_Frequency=1.0Hz<br>MassConc_1p0_SEN44_8E94_733F37AC73168E94<br><br>Device=SEN44_(1d6f75),Device_Serial_Number=SEN44-733F37AC73168E94,Port=0,SensorName=SEN44_8E94,Sensor_Serial_Number=733F37AC73168E94,Signal=Mass_Concentration_2p5,Type=float,Format=.6f,Unit=ug/m³,Product=SEN44,Measurement_Frequency=1.0Hz<br>MassConc_2p5_SEN44_8E94_733F37AC73168E94<br><br>Device=SEN44_(1d6f75),Device_Serial_Number=SEN44-733F37AC73168E94,Port=0,SensorName=SEN44_8E94,Sensor_Serial_Number=733F37AC73168E94,S

			ignal=Mass_Concentration_4p0,Type=float,Format=.6f,Unit=ug/m^3,Product=SEN44,Measurement_Frequency=1.0Hz<br>MassConc_4p0_SEN44_8E94_733F37AC73168E94<br><br>Device=SEN44_(1d6f75),Device_Serial_Number=SEN44-733F37AC73168E94,Port=0,SensorName=SEN44_8E94,Sensor_Serial_Number=733F37AC73168E94,Signal=Mass_Concentration_10p,Type=float,Format=.6f,Unit=ug/m^3,Product=SEN44,Measurement_Frequency=1.0Hz<br>MassConc_10p_SEN44_8E94_733F37AC73168E94<br><br>Device=SEN44_(1d6f75),Device_Serial_Number=SEN44-733F37AC73168E94,Port=0,SensorName=SEN44_8E94,Sensor_Serial_Number=733F37AC73168E94,Signal=Relative_Humidity,Type=float,Format=.6f,Unit=%RH,Product=SEN44,Measurement_Frequency=1.0Hz<br>RH_AMB_SEN44_8E94_733F37AC73168E94<br><br>Device=SEN44_(1d6f75),Device_Serial_Number=SEN44-733F37AC73168E94,Port=0,SensorName=SEN44_8E94,Sensor_Serial_Number=733F37AC73168E94,Signal=Temperature,Type=float,Format=.6f,Unit=°C,Product=SEN44,Measurement_Frequency=1.0Hz<br>T_AMB_SEN44_8E94_733F37AC73168E94<br><br>Device=SEN44_(1d6f75),Device_Serial_Number=SEN44-733F37AC73168E94,Port=0,SensorName=SEN44_8E94,Sensor_Serial_Number=733F37AC73168E94,Signal=VOC_Index,Type=float,Format=.6f,Unit=VOC Index,Product=SEN44,Measurement_Frequency=1.0Hz<br>Index_VOC_SEN44_8E94_733F37AC73168E94
Sensit Technologies (Valparaiso, IN, U.S.)	RAMP	No header, variable ID including in column before value	ID<br>Date<br>CO<br>NO<br>NO2<br>O3

			CO2<br>T<br>RH<br>PM1.0<br>PM2.5<br>PM10<br>WD<br>WS<br>BATT<br>CHRG<br>RUN<br>SD<br>RAW<br>STAT
TSI (Shoreview, MN, U.S.)	BlueSky	Row 1 header, Row 2 units	Timestamp<br>UTC<br>PM2.5<br>ug/m3<br>Applied PM2.5 Custom Calibration Setting - Multiplication Factor<br>PM10<br>ug/m3<br>Applied PM10 Custom Calibration Setting - Multiplication Factor<br>PM Sensor Status Temperature<br>Fahrenheit<br>Applied Temperature Custom Calibration Setting - Offset<br>Relative Humidity%<br>Applied Relative Humidity Custom Calibration Setting - Offset<br>Device Status
WA Department of Ecology (WA, U.S.)	Custom build with Sensiron[1]	No headers	Sensor ID and correction status<br>Date<br>Time<br>Concentration<br>Additional value
